# Nfs1 cysteine desulfurase protein complexes and phosphorylation sites as assessed by mass spectrometry

**DOI:** 10.1016/j.dib.2017.09.068

**Published:** 2017-10-06

**Authors:** Agostinho G. Rocha, Simon A.B. Knight, Alok Pandey, Heeyong Yoon, Jayashree Pain, Debkumar Pain, Andrew Dancis

**Affiliations:** a Department of Medicine, Division of Hematology-Oncology, Perelman School of Medicine, University of Pennsylvania, Philadelphia, PA, United States; b Department of Pharmacology, Physiology and Neuroscience, New Jersey Medical School, Rutgers University, Newark, NJ, United States

## Abstract

Fe-S clusters are cofactors that participate in diverse and essential biological processes. Mitochondria contain a complete machinery for Fe-S cluster assembly. Cysteine desulfurase (Nfs1) is required generation of a form of activated sulfur and is essential for the initial Fe-S cluster assembly step. Using mass-spectometry we identified proteins that were copurified with Nfs1 using a pull-down strategy, including a novel protein kinase. Furthermore, we were able to identify phosphorylation sites on the Nfs1 protein. These data and analyses support the research article “Cysteine desulfurase is regulated by phosphorylation of Nfs1 in yeast mitochondria” by Rocha et al. (in press) [1].

**Specifications Table**

**Table:** 

Subject area	Biology
More specific subject area	Mitochondrial Fe-S cluster biogenesis
Type of data	Mass spectrometry data
How data was acquired	Mass spectroscopy
Data format	Filtered and analyzed
Experimental factors	Recombinant and endogenous yeast Nfs1 cysteine desulfurase was purified under native conditions and further analyzed by LC-MS/MS
Experimental features	Various strategies were used to detect novel Nfs1 interacting proteins as well as phosphorylation sites
Data source location	Philadelphia, PA USA
Data accessibility	Data are accompanying this article

**Value of the data**

• LC-MS/MS was used to identify new proteins that associate with Nfs1 cysteine desulfurase and that potentially participate in the assembly of Fe-S clusters.
• Yck2, a protein kinase, was identified using independent approaches.
• Phosphorylation sites of Nfs1 were also identified using mass-spectrometric approaches.

## 1 Data

Pull-downs were performed from purified yeast mitochondria expressing Nfs1-His_6_ or Yfh1-His_6_ (Table 1).

**Table 1:** Complete list of proteins identified by MS/MS. Complete table of identified proteins, gene name and total MS/MS count are showed for the analysis of both protein complexes: Nfs1_His6_ and Yfh1_His6_.

**#**	**Identified Proteins on Nfs1-His6 sample**	**Gene names**	**Molecular Weight**	**Total MS/MS Count**
1	Cysteine desulfurase, mitochondrial	NFS1	54	1554
2	Alcohol dehydrogenase 3, mitochondrial	ADH3	40	1263
3	Elongation factor 1-?alpha	TEF1	50	340
4	Heat shock protein 60, mitochondrial	HSP60	61	452
5	V-type proton ATPase catalytic subunit A;Endonuclease PI-SceI	VMA1	119	365
6	Repressible alkaline phosphatase;Soluble alkaline phosphatase	PHO8	63	165
7	Threonine dehydratase, mitochondrial	ILV1	64	213
8	Peroxisomal acyl-coenzyme A thioester hydrolase 1	TES1	40	110
9	Myosin tail region-interacting protein MTI1	BBC1	128	105
10	Casein kinase I homolog 2	YCK2	62	164
11	Heat shock protein SSC1, mitochondrial	SSC1	71	132
12	Nicotinamidase	PNC1	25	87
13	Acetolactate synthase catalytic subunit, mitochondrial	ILV2	75	138
14	Cytochrome b-?c1 complex subunit 2, mitochondrial	QCR2	40	92
15	ATP synthase subunit beta, mitochondrial	ATP2	55	149
16	2-oxoglutarate dehydrogenase, mitochondrial	KGD1	114	138
17	Protein ISD11	ISD11	11	35
18	ATP synthase subunit alpha, mitochondrial	ATP1	59	104
19	Glyceraldehyde-3-phosphate dehydrogenase 3	TDH3	36	79
20	Ketol-acid reductoisomerase, mitochondrial	ILV5	44	102
21	Cytochrome b-?c1 complex subunit 1, mitochondrial	COR1	50	104
22	Mitochondrial outer membrane protein porin 1	POR1	30	75
23	Long-chain-fatty-acid--CoA ligase 1	FAA1	78	89
24	Alcohol dehydrogenase 2	ADH2	37	18
25	RNA-binding protein SRO9	SRO9	48	94
26	Carbon catabolite-derepressing protein kinase	SNF1	72	89
27	Glycerol-3-phosphate dehydrogenase [NAD(+)] 2, mitochondrial	GPD2	49	59
28	Potassium-activated aldehyde dehydrogenase, mitochondrial	ALD4	57	83
29	Altered inheritance of mitochondria protein 32	AIM32	36	64
30	Malate dehydrogenase, mitochondrial	MDH1	36	71
31	mRNA-binding protein PUF3	PUF3	98	48
32	Dihydrolipoyl dehydrogenase, mitochondrial	LPD1	54	95
33	Succinate dehydrogenase [ubiquinone] flavoprotein subunit, mitochondrial	SDH1	70	46
34	Serine hydroxymethyltransferase, mitochondrial	SHM1	54	70
35	Isocitrate dehydrogenase [NAD] subunit 2, mitochondrial	IDH2	40	47
36	Aconitate hydratase, mitochondrial	ACO1	85	88
37	Citrate synthase, mitochondrial	CIT1	53	65
38	Dihydrofolate synthetase	FOL3	48	62
39	Eukaryotic translation initiation factor 4E	CDC33	24	53
40	ADP,ATP carrier protein 2	PET9	34	48
41	2-isopropylmalate synthase	LEU4	68	70
42	Acetolactate synthase small subunit, mitochondrial	ILV6	34	83
43	Plasma membrane ATPase 1;Plasma membrane ATPase 2	PMA1;PMA2	100	83
44	Nucleoporin POM152	POM152	152	104
45	Altered inheritance of mitochondria protein 6	AIM6	44	60
46	Pyruvate kinase 1	CDC19	55	82
47	Branched-chain-amino-acid aminotransferase, mitochondrial	BAT1	44	54
48	Dihydroxy-acid dehydratase, mitochondrial	ILV3	63	70
49	37S ribosomal protein S5, mitochondrial	MRPS5	35	17
50	78 glucose-regulated protein homolog	KAR2	74	79
51	NAD-dependent malic enzyme, mitochondrial	MAE1	74	96
52	Elongation factor Tu, mitochondrial	TUF1	48	62
53	Isocitrate dehydrogenase [NAD] subunit 1, mitochondrial	IDH1	39	43
54	Homoisocitrate dehydrogenase, mitochondrial	LYS12	40	60
55	Clustered mitochondria protein 1	CLU1	145	95
56	Chromatin structure-remodeling complex protein RSC8	RSC8	63	57
57	External NADH-ubiquinone oxidoreductase 1, mitochondrial	NDE1	63	53
58	C-1-tetrahydrofolate synthase, mitochondrial	MIS1	106	69
59	Iron sulfur cluster assembly protein 2, mitochondrial	ISU2	17	37
60	Histone H2B.2;Histone H2B.1	HTB2;HTB1	14	18
61	Phosphatidylethanolamine N-?methyltransferase	CHO2	101	38
62	Uncharacterized WD repeat-containing protein YOL087C		125	68
63	Nucleolar protein 3	NPL3	45	35
64	Serine/threonine-protein kinase HRK1	HRK1	86	48
65	Protein RMD9-like, mitochondrial		84	60
66	Nicotinamide-nucleotide adenylyltransferase 1	NMA1	46	50
67	ATP-dependent RNA helicase MSS116, mitochondrial	MSS116	76	61
68	Uncharacterized protein YGR266W	YGR266W	81	53
69	Pyruvate dehydrogenase E1 component subunit alpha, mitochondrial	PDA1	46	46
70	Lon protease homolog, mitochondrial	PIM1	127	83
71	Transposon Ty1-NL2 Gag-Pol polyprotein;Capsid protein	TY1B-NL2	198	51
72	Dihydrolipoyllysine-residue succinyltransferase component of 2-?oxoglutarate dehydrogenase complex, mitochondrial	KGD2	50	28
73	Alcohol dehydrogenase 1	ADH1	37	19
74	tRNA N6-adenosine threonylcarbamoyltransferase, mitochondrial	QRI7	46	36
75	5-AMP-activated protein kinase subunit gamma	SNF4	36	50
76	Chromatin structure-remodeling complex protein RSC6	RSC6	54	34
77	Pyruvate decarboxylase isozyme 1	PDC1	61	47
78	54S ribosomal protein YmL6, mitochondrial	YML6	32	35
79	Putative transcription factor SEF1	SEF1	128	22
80	Nuclear protein STH1/NPS1	STH1	157	57
81	AMP deaminase	AMD1	93	48
82	37S ribosomal protein S23, mitochondrial	RSM23	51	44
83	Enhancer of polycomb-like protein 1	EPL1	97	58
84	Iron sulfur cluster assembly protein 1, mitochondrial	ISU1	18	18
85	Cytochrome b2, mitochondrial	CYB2	66	30
86	Heat shock protein SSA1	SSA1	70	56
87	Mitochondrial acidic protein MAM33	MAM33	30	22
88	Pyruvate dehydrogenase E1 component subunit beta, mitochondrial	PDB1	40	37
89	ATP synthase subunit gamma, mitochondrial	ATP3	34	32
90	Homoaconitase, mitochondrial	LYS4	75	53
91	Protein disulfide-isomerase	PDI1	58	45
92	Isocitrate dehydrogenase [NADP], mitochondrial	IDP1	48	46
93	Chromatin structure-remodeling complex subunit RSC9	RSC9	65	38
94	GTP cyclohydrolase 1	FOL2	28	30
95	Putative pterin-4-alpha-carbinolamine dehydratase	YHL018W	14	15
96	D-lactate dehydrogenase [cytochrome] 1, mitochondrial	DLD1	65	34
97	Heat shock protein SSB1	SSB1	67	32
98	Protein MMF1, mitochondrial	MMF1	16	16
99	DnaJ homolog 1, mitochondrial	MDJ1	56	44
100	Elongation factor 3A	YEF3	116	48
101	Acetyl-CoA hydrolase	ACH1	59	50
102	Fumarate hydratase, mitochondrial	FUM1	53	32
103	ATP-dependent RNA helicase DED1	DED1	66	50
104	Succinate dehydrogenase [ubiquinone] iron-sulfur subunit, mitochondrial	SDH2	30	24
105	Chromatin structure-remodeling complex subunit SFH1	SFH1	49	27
106	Dihydrolipoyllysine-residue acetyltransferase component of pyruvate dehydrogenase complex, mitochondrial	LAT1	52	30
107	Vacuolar protein sorting-associated protein 1	VPS1	79	45
108	Putative glucokinase-2	EMI2	56	36
109	Succinyl-CoA ligase [ADP-forming] subunit beta, mitochondrial	LSC2	47	35
110	Protein URA2;Glutamine-dependent carbamoyl-phosphate synthase	URA2	245	31
111	Aldehyde dehydrogenase 5, mitochondrial	ALD5	57	29
112	Protein OPY1	OPY1	38	35
113	37S ribosomal protein MRP4, mitochondrial	MRP4	44	25
114	Probable oxidoreductase AIM17	AIM17	53	25
115	Putative 2-?hydroxyacid dehydrogenase YPL113C	YPL113C	45	40
116	UTP--glucose-1-phosphate uridylyltransferase	UGP1	56	30
117	Protein PSP2	PSP2	66	26
118	Phosphoglycerate kinase	PGK1	45	34
119	37S ribosomal protein MRP1, mitochondrial	MRP1	37	21
120	Mitochondrial phosphate carrier protein	MIR1	33	26
121	Succinyl-CoA ligase [ADP-forming] subunit alpha, mitochondrial	LSC1	35	25
122	Catabolic L-?serine/threonine dehydratase	CHA1	39	28
123	37S ribosomal protein S35, mitochondrial	MRPS35	40	30
124	Enolase 2	ENO2	47	30
125	54S ribosomal protein L13, mitochondrial	MRPL13	30	20
126	Protein AST1	AST1	48	25
127	60S ribosomal protein L4-A;60S ribosomal protein L4-B	RPL4A;RPL4B	39	23
128	Uncharacterized RNA-binding protein YGR250C	YGR250C	90	37
129	54S ribosomal protein L3, mitochondrial	MRPL3	44	24
130	37S ribosomal protein NAM9, mitochondrial	NAM9	56	36
131	Actin	ACT1	42	28
132	Inorganic pyrophosphatase	IPP1	32	28
133	Mitochondrial metal transporter 2	MMT2	52	20
134	Glycerol-3-phosphate dehydrogenase, mitochondrial	GUT2	72	44
135	Carboxypeptidase S	CPS1	65	30
136	Protein kinase-like protein SCY1	SCY1	91	43
137	Serine hydroxymethyltransferase, cytosolic	SHM2	52	33
138	Ornithine aminotransferase	CAR2	46	28
139	Acetylornithine aminotransferase, mitochondrial	ARG8	47	27
140	Flavohemoprotein	YHB1	45	26
141	Probable alanine aminotransferase, mitochondrial	ALT1	66	24
142	Actin-like protein ARP9	ARP9	53	25
143	Homocitrate dehydratase, mitochondrial	ACO2	87	30
144	54S ribosomal protein L4, mitochondrial	MRPL4	37	26
145	Histone H4	HHF1	11	23
146	Actin patches distal protein 1	APD1	36	28
147	54S ribosomal protein L35, mitochondrial	MRPL35	43	31
148	Protein ARG5,6, mitochondrial	ARG5,6	95	40
149	Mitochondrial presequence protease	CYM1	112	41
150	37S ribosomal protein S24, mitochondrial	RSM24	37	38
151	Heat shock protein 78, mitochondrial	HSP78	91	41
152	54S ribosomal protein L40, mitochondrial	MRPL40	34	23
153	Mitochondrial peroxiredoxin PRX1	PRX1	29	16
154	Pyruvate dehydrogenase complex protein X component, mitochondrial	PDX1	45	22
155	GTPase MTG2, mitochondrial	MTG2	58	26
156	37S ribosomal protein MRP51, mitochondrial	MRP51	39	24
157	Cell division control protein 12	CDC12	47	26
158	Cell division control protein 3	CDC3	60	22
159	Probable serine/threonine-protein kinase KKQ8	KKQ8	83	29
160	Glucokinase-1	GLK1	55	27
161	40S ribosomal protein S4-B;40S ribosomal protein S4-A	RPS4B;RPS4A	29	21
162	Histone acetyltransferase ESA1	ESA1	53	30
163	GrpE protein homolog, mitochondrial	MGE1	26	16
164	Chromatin structure-remodeling complex protein RSC58	RSC58	58	36
165	Histone H3	HHT1	15	5
166	Acetyl-CoA carboxylase;Biotin carboxylase	ACC1	250	63
167	RuvB-like protein 1	RVB1	50	27
168	Homocitrate synthase, cytosolic isozyme	LYS20	47	26
169	Altered inheritance of mitochondria protein 9, mitochondrial	AIM9	72	32
170	Cytochrome c1, heme protein, mitochondrial	CYT1	34	15
171	37S ribosomal protein S28, mitochondrial	MRPS28	33	20
172	40S ribosomal protein S5	RPS5	25	24
173	Sphingolipid long chain base-responsive protein PIL1	PIL1	38	24
174	37S ribosomal protein S25, mitochondrial	RSM25	31	20
175	37S ribosomal protein PET123, mitochondrial	PET123	36	21
176	Glyceraldehyde-3-phosphate dehydrogenase 1	TDH1	36	20
177	Replication factor C subunit 1	RFC1	95	5
178	60S ribosomal protein L5	RPL5	34	17
179	(R,R)-butanediol dehydrogenase	BDH1	42	21
180	40S ribosomal protein S3	RPS3	27	21
181	Nicotinamide-nucleotide adenylyltransferase 2	NMA2	45	24
182	RuvB-like protein 2	RVB2	52	25
183	Chromatin structure-remodeling complex subunit RSC7	NPL6	50	13
184	Serine/threonine-protein kinase YPK2/YKR2	YPK2	77	30
185	Rotenone-insensitive NADH-ubiquinone oxidoreductase, mitochondrial	NDI1	57	22
186	40S ribosomal protein S1-B	RPS1B	29	15
187	Hexokinase-1	HXK1	54	31
188	60S acidic ribosomal protein P0	RPP0	34	16
189	Heat shock protein 42	HSP42	43	18
190	Putative Xaa-Pro aminopeptidase FRA1	FRA1	85	26
191	Prohibitin-1	PHB1	31	20
192	54S ribosomal protein L1, mitochondrial	MRPL1	31	18
193	54S ribosomal protein L2, mitochondrial	MRP7	43	27
194	3-hydroxy-3-methylglutaryl-coenzyme A reductase 1	HMG1	116	25
195	Mitochondrial genome maintenance protein MGM101	MGM101	30	21
196	Chromatin structure-remodeling complex subunit RSC2	RSC2	102	19
197	37S ribosomal protein S7, mitochondrial	RSM7	28	21
198	60S ribosomal protein L8-B;60S ribosomal protein L8-A	RPL8B;RPL8A	28	26
199	ATP synthase subunit 4, mitochondrial	ATP4	27	12
200	Protein dopey	DOP1	195	47
201	Aminomethyltransferase, mitochondrial	GCV1	44	21
202	54S ribosomal protein L20, mitochondrial	MRPL20	22	14
203	Galactokinase	GAL1	58	20
204	Cell division control protein 10	CDC10	37	17
205	Sterol 24-C-methyltransferase	ERG6	43	24
206	Uncharacterized protein YBL086C		52	7
207	Actin-related protein 7	ARP7	54	19
208	Myosin-3	MYO3	142	21
209	Ribosomal protein VAR1, mitochondrial	VAR1	47	22
210	Glycine cleavage system H protein, mitochondrial	GCV3	19	12
211	NADH-cytochrome b5 reductase 2	MCR1	34	13
212	Uncharacterized protein YHR097C	YHR097C	41	11
213	Mitochondrial intermediate peptidase	OCT1	88	31
214	37S ribosomal protein MRP13, mitochondrial	MRP13	39	18
215	ATP synthase subunit 5, mitochondrial	ATP5	23	13
216	60S ribosomal protein L3	RPL3	44	20
217	Protein SCP160	SCP160	135	34
218	Zinc/cadmium resistance protein	ZRC1	48	9
219	Enoyl-[acyl-carrier protein] reductase [NADPH, B-?specific], mitochondrial	ETR1	42	25
220	60S ribosomal protein L2-B;60S ribosomal protein L2-A	RPL2B;RPL2A	27	12
221	Inhibitory regulator protein IRA1	IRA1	351	38
222	Mitochondrial-processing peptidase subunit alpha	MAS2	53	24
223	PAB1-binding protein 1	PBP1	79	14
224	Inhibitory regulator protein IRA2	IRA2	352	35
225	Cytochrome c oxidase subunit 4, mitochondrial	COX4	17	12
226	5-aminolevulinate synthase, mitochondrial	HEM1	59	22
227	Protein GTS1	GTS1	44	23
228	40S ribosomal protein S20	RPS20	14	11
229	Something about silencing protein 4	SAS4	55	3
230	Long-chain-fatty-acid--CoA ligase 4	FAA4	77	17
231	54S ribosomal protein L8, mitochondrial	MRPL8	27	13
232	54S ribosomal protein L15, mitochondrial	MRPL15	28	9
233	40S ribosomal protein S0-B;40S ribosomal protein S0-A	RPS0B;RPS0A	28	12
234	Mitochondrial protein import protein MAS5	YDJ1	45	25
235	Ferrochelatase, mitochondrial	HEM15	45	23
236	37S ribosomal protein RSM28, mitochondrial	RSM28	41	16
237	SNF1 protein kinase subunit beta-2	SIP2	46	16
238	Factor arrest protein 8	FAR8	59	11
239	40S ribosomal protein S2	RPS2	27	8
240	ARS-binding factor 2, mitochondrial	ABF2	22	10
241	37S ribosomal protein S9, mitochondrial	MRPS9	32	9
242	Phosphoglycerate mutase 1	GPM1	28	19
243	37S ribosomal protein S10, mitochondrial	RSM10	23	7
244	Serine/threonine-protein kinase KIN2	KIN2	128	21
245	37S ribosomal protein S17, mitochondrial	MRPS17	28	12
246	54S ribosomal protein L9, mitochondrial	MRPL9	30	10
247	Vacuolar aminopeptidase 1	LAP4	57	19
248	2-methoxy-6-polyprenyl-1,4-benzoquinol methylase, mitochondrial	COQ5	35	14
249	Mitochondrial metal transporter 1	MMT1	56	21
250	Cell division control protein 11	CDC11	48	13
251	Single-stranded DNA-binding protein RIM1, mitochondrial	RIM1	15	7
252	DnaJ-related protein SCJ1	SCJ1	42	14
253	ATP-dependent 6-?phosphofructokinase subunit alpha	PFK1	108	28
254	Histone H2A.Z	HTZ1	14	7
255	Chromatin structure-remodeling complex protein RSC3	RSC3	102	22
256	Prohibitin-2	PHB2	34	16
257	Galactose-1-phosphate uridylyltransferase	GAL7	42	15
258	Superoxide dismutase [Mn], mitochondrial	SOD2	26	11
259	RHO1 GDP-GTP exchange protein 2	ROM2	153	28
260	54S ribosomal protein L7, mitochondrial	MRPL7	33	16
261	Single-stranded nucleic acid-binding protein	SBP1	33	13
262	Ubiquitin carboxyl-terminal hydrolase 3	UBP3	102	6
263	Saccharopepsin	PEP4	44	14
264	Nucleolar protein 58	NOP58	57	17
265	Uncharacterized protein YNL011C		50	20
266	Meiotic sister-chromatid recombination protein 6, mitochondrial	MSC6	80	23
267	Mitochondrial import receptor subunit TOM70	TOM70	70	22
268	40S ribosomal protein S9-B;40S ribosomal protein S9-A	RPS9B;RPS9A	22	8
269	Cargo-transport protein YPP1	YPP1	95	20
270	Mitochondrial import receptor subunit TOM40	TOM40	42	10
271	Glutamate decarboxylase	GAD1	66	24
272	Squalene synthase	ERG9	52	16
273	Lysine--tRNA ligase, mitochondrial	MSK1	66	16
274	37S ribosomal protein S22, mitochondrial	RSM22	72	21
275	54S ribosomal protein L17, mitochondrial	MRPL17	32	11
276	Chaotic nuclear migration protein 67	CNM67	67	21
277	54S ribosomal protein L12, mitochondrial	MNP1	21	7
278	Delta-1-pyrroline-5-carboxylate dehydrogenase, mitochondrial	PUT2	64	14
279	40S ribosomal protein S6-B;40S ribosomal protein S6-A	RPS6B;RPS6A	27	12
280	Elongation factor 2	EFT1	93	22
281	DNA-directed RNA polymerase, mitochondrial	RPO41	153	26
282	Protein AFG1	AFG1	58	16
283	54S ribosomal protein L22, mitochondrial	MRPL22	35	18
284	Acyl carrier protein, mitochondrial	ACP1	14	5
285	High-affinity hexose transporter HXT6	HXT7;HXT6	63	9
286	Proteasome activator BLM10	BLM10	246	3
287	Cerevisin	PRB1	70	11
288	GTPase-activating protein BEM2/IPL2	BEM2	245	27
289	Lanosterol 14-alpha demethylase	ERG11	61	21
290	Small COPII coat GTPase SAR1	SAR1	21	11
291	60S ribosomal protein L7-A;60S ribosomal protein L7-B	RPL7A;RPL7B	28	14
292	Morphogenesis-related protein MSB1	MSB1	130	21
293	Mitochondrial peculiar membrane protein 1	MPM1	28	15
294	Hexokinase-2	HXK2	54	21
295	Guanine nucleotide-binding protein subunit beta-like protein	ASC1	35	11
296	Protein MSS51, mitochondrial	MSS51	51	15
297	Fructose-bisphosphate aldolase	FBA1	40	13
298	Bifunctional protein GAL10;UDP-glucose 4-?epimerase;Aldose 1-?epimerase	GAL10	78	16
299	Phosphatidylinositol 4-?kinase STT4	STT4	215	24
300	54S ribosomal protein L10, mitochondrial	MRPL10	36	6
301	Mitochondrial-processing peptidase subunit beta	MAS1	51	11
302	ISWI chromatin-remodeling complex ATPase ISW1	ISW1	131	20
303	NifU-like protein, mitochondrial	NFU1	29	10
304	40S ribosomal protein S7-A	RPS7A	22	14
305	Biotin synthase, mitochondrial	BIO2	42	15
306	Ubiquitin carboxyl-terminal hydrolase 11	UBP11	83	17
307	Probable quinone oxidoreductase	ZTA1	37	5
308	ATP-dependent RNA helicase eIF4A	TIF1	45	16
309	Fatty acid synthase subunit alpha	FAS2	207	23
310	Arginine biosynthesis bifunctional protein ArgJ	ARG7	48	18
311	[Pyruvate dehydrogenase (acetyl-transferring)] kinase 2, mitochondrial	PKP2	57	17
312	MICOS complex subunit MIC60	MIC60	61	19
313	Factor arrest protein 7	FAR7	26	13
314	Squalene monooxygenase	ERG1	55	7
315	Dual specificity protein phosphatase PPS1	PPS1	92	14
316	Phosphatidylinositol 4,5-bisphosphate 5-?phosphatase INP51	INP51	108	14
317	Catabolite repression protein CAT5	CAT5	26	9
318	Cytochrome b-?c1 complex subunit Rieske, mitochondrial	RIP1	23	10
319	37S ribosomal protein S26, mitochondrial	RSM26	30	9
320	5-3 exoribonuclease 1	XRN1	175	17
321	Serine--tRNA ligase, cytoplasmic	SES1	53	12
322	54S ribosomal protein L41, mitochondrial	MRP20	31	9
323	Mediator of RNA polymerase II transcription subunit 13	SSN2	160	16
324	General negative regulator of transcription subunit 4	MOT2	65	5
325	ATP-dependent 6-?phosphofructokinase subunit beta	PFK2	105	19
326	Mitochondrial DNA replication protein YHM2	YHM2	34	8
327	Nucleolar protein 56	NOP56	57	20
328	Fatty acid synthase subunit beta	FAS1	229	23
329	Aminopeptidase 2, mitochondrial	APE2	108	16
330	Sphingolipid long chain base-responsive protein LSP1	LSP1	38	15
331	ARS-binding factor 1	ABF1	82	8
332	Glycine dehydrogenase (decarboxylating), mitochondrial	GCV2	114	17
333	tRNA-aminoacylation cofactor ARC1	ARC1	42	8
334	[Pyruvate dehydrogenase (acetyl-transferring)] kinase 1, mitochondrial	PKP1	45	9
335	NAD(+) kinase	UTR1	59	13
336	Polyadenylate-binding protein, cytoplasmic and nuclear	PAB1	64	11
337	Protein RMD9, mitochondrial	RMD9	75	17
338	SNF1 protein kinase subunit beta-1	SIP1	91	11
339	Peptidyl-prolyl cis-trans isomerase C, mitochondrial	CPR3	20	9
340	60S ribosomal protein L16-A	RPL16A	22	5
341	Vesicular-fusion protein SEC18	SEC18	84	19
342	Inosine-5-monophosphate dehydrogenase 3 ;Inosine-5-monophosphate dehydrogenase 4	IMD3;IMD4	57	15
343	Ubiquitin-60S ribosomal protein L40	RPL40B;RPL40A;RPS31;UBI4	15	3
344	37S ribosomal protein S8, mitochondrial	MRPS8	17	7
345	Target of rapamycin complex subunit LST8	LST8	34	14
346	cAMP-dependent protein kinase regulatory subunit	BCY1	47	11
347	Probable electron transfer flavoprotein subunit alpha, mitochondrial	AIM45	37	12
348	Glyceraldehyde-3-phosphate dehydrogenase 2	TDH2	36	4
349	Malate dehydrogenase, peroxisomal	MDH3	37	16
350	2-isopropylmalate synthase 2, mitochondrial	LEU9	67	15
351	Protein transport protein SEC23	SEC23	85	14
352	Chromatin structure-remodeling complex subunit RSC4	RSC4	72	9
353	Cytochrome c peroxidase, mitochondrial	CCP1	40	12
354	Cell division control protein 48	CDC48	92	20
355	Ubiquinone biosynthesis protein COQ9, mitochondrial	COQ9	30	6
356	Lipoyl synthase, mitochondrial	LIP5	46	13
357	Mitochondrial chaperone TCM62	TCM62	64	11
358	6-phosphogluconate dehydrogenase, decarboxylating 1	GND1	54	16
359	Inorganic pyrophosphatase, mitochondrial	PPA2	36	10
360	Heat shock protein SSQ1, mitochondrial	SSQ1	72	13
361	Protein ATP12, mitochondrial	ATP12	37	9
362	V-type proton ATPase subunit B	VMA2	58	21
363	60S ribosomal protein L9-A;60S ribosomal protein L9-B	RPL9A;RPL9B	22	8
364	Regulatory protein ADR1	ADR1	151	15
365	NADPH--cytochrome P450 reductase	NCP1	77	16
366	UBP3-associated protein BRE5	BRE5	58	10
367	Sigma-like sequence protein 1, mitochondrial	SLS1	73	14
368	Elongation factor G, mitochondrial	MEF1	85	11
369	Factor arrest protein 3	FAR3	24	10
370	GTP-binding protein RHO1	RHO1	23	5
371	60S ribosomal protein L13-A;60S ribosomal protein L13-B	RPL13A;RPL13B	23	7
372	Mediator of RNA polymerase II transcription subunit 12	SRB8	167	15
373	Probable electron transfer flavoprotein-ubiquinone oxidoreductase, mitochondrial	CIR2	70	16
374	Aminopeptidase Y	APE3	60	6
375	T-complex protein 1?subunit theta	CCT8	62	9
376	Ribonuclease P protein component, mitochondrial	RPM2	139	19
377	Protein SOF1	SOF1	57	11
378	Nuclear segregation protein BFR1	BFR1	55	17
379	Transcription elongation factor SPT6	SPT6	168	15
380	WD repeat-containing protein YMR102C		94	11
381	Mitochondrial inner membrane i-?AAA protease supercomplex subunit YME1	YME1	82	14
382	Mitochondrial import inner membrane translocase subunit TIM44	TIM44	49	12
383	Sorting nexin-4	SNX4	49	11
384	Ubiquitin ligase-binding protein BUL1	BUL1	109	15
385	54S ribosomal protein L11, mitochondrial	MRPL11	29	9
386	Protein MON2	MON2	187	16
387	V-type proton ATPase subunit a, vacuolar isoform	VPH1	96	15
388	D-arabinono-1,4-lactone oxidase	ALO1	59	10
389	Cobalt uptake protein COT1	COT1	48	7
390	Dolichol-phosphate mannosyltransferase	DPM1	30	11
391	60S ribosomal protein L17-B;60S ribosomal protein L17-A	RPL17B;RPL17A	21	3
392	Tryptophan--tRNA ligase, mitochondrial	MSW1	43	12
393	Probable serine/threonine-protein kinase RTK1	RTK1	70	8
394	3-keto-steroid reductase	ERG27	40	11
395	Mitochondrial homologous recombination protein 1	MHR1	27	6
396	T-complex protein 1?subunit beta	CCT2	57	16
397	Leucine--tRNA ligase, mitochondrial	NAM2	102	10
398	40S ribosomal protein S18-B;40S ribosomal protein S18-A	RPS18B;RPS18A	17	5
399	Fatty aldehyde dehydrogenase HFD1	HFD1	60	9
400	Guanine nucleotide-binding protein subunit beta 1	GPB1	101	14
401	ATP-dependent molecular chaperone HSC82;ATP-dependent molecular chaperone HSP82	HSC82;HSP82	81	15
402	Glutamyl-tRNA(Gln) amidotransferase subunit B, mitochondrial	PET112	62	11
403	C-8 sterol isomerase	ERG2	25	5
404	Protein transport protein SEC24	SEC24	104	15
405	60S ribosomal protein L19-B;60S ribosomal protein L19-A	RPL19B;RPL19A	22	6
406	54S ribosomal protein L24, mitochondrial	MRPL24	30	9
407	60S ribosomal protein L11-B;60S ribosomal protein L11-A	RPL11B;RPL11A	20	5
408	Intracellular protein transport protein USO1	USO1	206	4
409	Ubiquinone biosynthesis protein COQ4, mitochondrial	COQ4	39	9
410	Protein phosphatase 2C homolog 4	PTC4	44	11
411	Hexaprenyl pyrophosphate synthase, mitochondrial	COQ1	53	9
412	Tricalbin-3	TCB3	171	6
413	Uncharacterized protein YLL007C		77	16
414	D-lactate dehydrogenase [cytochrome] 2, mitochondrial	DLD2	59	14
415	Protein SIS1	SIS1	38	8
416	Transcription-associated protein 1	TRA1	433	22
417	DNA topoisomerase 2	TOP2	164	18
418	Mitochondrial oxaloacetate transport protein	OAC1	35	7
419	Cytochrome B pre-mRNA-processing protein 2	CBP2	74	9
420	40S ribosomal protein S11-B;40S ribosomal protein S11-A	RPS11B;RPS11A	18	6
421	60S ribosomal protein L6-A	RPL6A	20	10
422	Meiotic mRNA stability protein kinase SSN3	SSN3	63	8
423	Reticulon-like protein 1	RTN1	33	8
424	54S ribosomal protein L51, mitochondrial	MRPL51	16	7
425	Protein phosphatase 1?regulatory subunit SDS22	SDS22	39	13
426	Protein TMA108	TMA108	108	11
427	Ribonucleoside-diphosphate reductase small chain 1	RNR2	46	12
428	Oxysterol-binding protein homolog 3	OSH3	114	12
429	Phosphatidylinositol transfer protein PDR16	PDR16	41	11
430	40S ribosomal protein S15	RPS15	16	4
431	Amidophosphoribosyltransferase	ADE4	57	12
432	Vacuolar protein sorting-associated protein 13	VPS13	358	14
433	NADH kinase POS5, mitochondrial	POS5	46	10
434	Probable electron transfer flavoprotein subunit beta	CIR1	29	6
435	Asparagine--tRNA ligase, mitochondrial	SLM5	57	7
436	Putative metallocarboxypeptidase ECM14	ECM14	50	5
437	60S ribosomal protein L10	RPL10	25	5
438	UPF0061 protein FMP40	FMP40	78	15
439	Mitochondrial respiratory chain complexes assembly protein AFG3	AFG3	85	8
440	60S ribosomal protein L15-A;60S ribosomal protein L15-B	RPL15A;RPL15B	24	6
441	60S ribosomal protein L12-B;60S ribosomal protein L12-A	RPL12B;RPL12A	18	7
442	Chromatin modification-related protein YNG2	YNG2	32	4
443	60S ribosomal protein L24-B;60S ribosomal protein L24-A	RPL24B;RPL24A	18	3
444	Uncharacterized protein YKR070W	YKR070W	39	5
445	Eukaryotic translation initiation factor 5A-1	HYP2	17	13
446	Glycine--tRNA ligase 1, mitochondrial	GRS1	75	13
447	Saccharopine dehydrogenase [NAD(+), L-?lysine-forming]	LYS1	41	10
448	Glucose-6-phosphate isomerase	PGI1	61	11
449	54S ribosomal protein IMG1, mitochondrial	IMG1	19	10
450	Peroxisomal-coenzyme A synthetase	PCS60	60	9
451	40S ribosomal protein S1-A	RPS1A	29	3
452	Serine/threonine-protein kinase TOR1	TOR1	281	6
453	Sterol-4-alpha-carboxylate 3-?dehydrogenase, decarboxylating	ERG26	39	6
454	Phosphoinositide phosphatase SAC1	SAC1	71	14
455	Superoxide dismutase [Cu-Zn]	SOD1	16	6
456	40S ribosomal protein S14-A;40S ribosomal protein S14-B	RPS14A;RPS14B	15	2
457	60S ribosomal protein L18-B;60S ribosomal protein L18-A	RPL18B;RPL18A	21	7
458	Nuclear localization sequence-binding protein	NSR1	45	5
459	Ras-related protein SEC4	SEC4	24	6
460	Low affinity vacuolar monovalent cation/H(+) antiporter	VNX1	103	7
461	40S ribosomal protein S26-B;40S ribosomal protein S26-A	RPS26B;RPS26A	13	4
462	Protein kinase MCK1	MCK1	43	9
463	Vacuolar protein 8	VAC8	63	10
464	T-complex protein 1?subunit eta	CCT7	60	8
465	60S ribosomal protein L26-B;60S ribosomal protein L26-A	RPL26B;RPL26A	14	6
466	Intrastrand cross-link recognition protein	IXR1	68	8
467	40S ribosomal protein S17-B;40S ribosomal protein S17-A	RPS17B;RPS17A	16	5
468	Uncharacterized protein YER077C	YER077C	80	9
469	Methionyl-tRNA formyltransferase, mitochondrial	FMT1	45	10
470	6,7-dimethyl-8-ribityllumazine synthase	RIB4	19	5
471	Cytochrome c iso-1	CYC1	12	9
472	Eukaryotic peptide chain release factor subunit 1	SUP45	49	9
473	Protein ATP11, mitochondrial	ATP11	37	8
474	Carnitine O-?acetyltransferase, mitochondrial	CAT2	75	9
475	Chromatin structure-remodeling complex protein RSC30	RSC30	101	4
476	Mitochondrial RNA-splicing protein MRS1	MRS1	41	8
477	Medium-chain fatty acid ethyl ester synthase/esterase 2	EHT1	51	8
478	Dolichyl-diphosphooligosaccharide--protein glycosyltransferase subunit WBP1	WBP1	49	7
479	Homocitrate synthase, mitochondrial	LYS21	49	5
480	SNF1 protein kinase subunit beta-3	GAL83	47	6
481	[Pyruvate dehydrogenase [acetyl-transferring]]-phosphatase 1, mitochondrial	PTC5	64	8
482	Nitrogen permease regulator 2	NPR2	70	6
483	Regulator of Ty1 transposition protein 102	RTT102	18	6
484	Translation initiation factor IF-2, mitochondrial	IFM1	76	8
485	Carboxypeptidase Y	PRC1	60	5
486	54S ribosomal protein L28, mitochondrial	MRPL28	17	5
487	Peroxiredoxin TSA1	TSA1	22	6
488	Trehalose synthase complex regulatory subunit TPS3	TPS3	119	10
489	40S ribosomal protein S13	RPS13	17	6
490	Mitochondrial GTP/GDP carrier protein 1	GGC1	33	8
491	NADPH-dependent 1-?acyldihydroxyacetone phosphate reductase	AYR1	33	6
492	Sphingosine-1-phosphate lyase	DPL1	66	6
493	Valine--tRNA ligase, mitochondrial	VAS1	120	10
494	Galactose transporter	GAL2	64	7
495	Cytochrome c oxidase subunit 2	COX2	29	4
496	3-methyl-2-oxobutanoate hydroxymethyltransferase	ECM31	34	7
497	Chromatin structure-remodeling complex protein RSC14	LDB7	20	4
498	Cap-associated protein CAF20	CAF20	18	5
499	Partitioning protein REP1	REP1	43	5
500	40S ribosomal protein S8-B;40S ribosomal protein S8-A	RPS8B;RPS8A	22	2
501	Aspartate--tRNA ligase, mitochondrial	MSD1	75	10
502	Aspartate--tRNA ligase, cytoplasmic	DPS1	64	10
503	Protein GVP36	GVP36	37	7
504	Sister chromatid cohesion protein PDS5	PDS5	147	9
505	Eukaryotic translation initiation factor 4B	TIF3	49	7
506	Sorting nexin-3	SNX3	19	6
507	Iron-sulfur clusters transporter ATM1, mitochondrial	ATM1	78	3
508	Mitochondrial group I intron splicing factor CCM1	CCM1	101	9
509	Triosephosphate isomerase	TPI1	27	6
510	54S ribosomal protein L23, mitochondrial	MRPL23	18	4
511	ATP synthase subunit d, mitochondrial	ATP7	20	4
512	Inorganic phosphate transport protein PHO88	PHO88	21	3
513	T-complex protein 1?subunit alpha	TCP1	60	9
514	Tyrosine-protein phosphatase CDC14	CDC14	62	8
515	Heat shock protein SSA4	SSA4	70	4
516	Factor arrest protein 10	FAR10	54	5
517	RNA-binding protein SGN1	SGN1	29	8
518	3-hydroxyisobutyryl-CoA hydrolase, mitochondrial	EHD3	56	7
519	Mitochondrial import inner membrane translocase subunit TIM50	TIM50	55	6
520	Monothiol glutaredoxin-5, mitochondrial	GRX5	17	6
521	LETM1 domain-containing protein YLH47, mitochondrial	YLH47	52	4
522	Phospho-2-dehydro-3-deoxyheptonate aldolase, tyrosine-inhibited	ARO4	40	3
523	Genetic interactor of prohibitins 3, mitochondrial	GEP3	64	9
524	ADP-ribosylation factor 1	ARF1	21	6
525	54S ribosomal protein L19, mitochondrial	MRPL19	17	5
526	60S ribosomal protein L25	RPL25	16	5
527	Guanine nucleotide exchange factor SRM1	SRM1	53	6
528	Clathrin coat assembly protein AP180B	YAP1802	64	5
529	Nucleoporin NIC96	NIC96	96	9
530	Acetyl-coenzyme A synthetase 2	ACS2	75	13
531	Cysteine proteinase 1, mitochondrial	LAP3	52	6
532	ATP-dependent RNA helicase DHH1	DHH1	58	6
533	T-complex protein 1?subunit delta	CCT4	58	8
534	D-lactate dehydrogenase [cytochrome] 3	DLD3	55	11
535	40S ribosomal protein S7-B	RPS7B	22	4
536	Protein EMP47	EMP47	50	6
537	Protein CBP3, mitochondrial	CBP3	39	8
538	60S ribosomal protein L20-B;60S ribosomal protein L20-A	RPL20B;RPL20A	20	4
539	DOCK-like protein YLR422W	YLR422W	222	9
540	V-type proton ATPase subunit d	VMA6	40	3
541	37S ribosomal protein S18, mitochondrial	MRPS18	25	3
542	Obg-like ATPase 1	OLA1	44	7
543	Glutamyl-tRNA(Gln) amidotransferase subunit A, mitochondrial	HER2	51	4
544	Replication factor C subunit 2	RFC2	40	7
545	Mitochondrial transcription factor 2	MTF2	51	7
546	LAS seventeen-binding protein 3	LSB3	49	4
547	Transcription initiation factor TFIID subunit 6	TAF6	58	5
548	Mitochondrial translation optimization protein 1	MTO1	74	8
549	Vacuolar transporter chaperone 4	VTC4	83	7
550	GTP-binding protein YPT7	YPT7	23	4
551	Asparagine synthetase [glutamine-hydrolyzing] 2 ;Asparagine synthetase [glutamine-hydrolyzing] 1	ASN2;ASN1	65	7
552	Ubiquinone biosynthesis monooxygenase COQ6	COQ6	54	6
553	37S ribosomal protein MRP21, mitochondrial	MRP21	20	5
554	Putative zinc metalloproteinase YIL108W	YIL108W	77	8
555	60S ribosomal protein L14-B;60S ribosomal protein L14-A	RPL14B;RPL14A	15	3
556	Actin-related protein 5	ARP5	88	9
557	40S ribosomal protein S24-B;40S ribosomal protein S24-A	RPS24B;RPS24A	15	5
558	60S ribosomal protein L28	RPL28	17	3
559	Thioredoxin-3, mitochondrial	TRX3	14	5
560	Protein BMH1;Protein BMH2	BMH1;BMH2	30	4
561	40S ribosomal protein S22-B;40S ribosomal protein S22-A	RPS22B;RPS22A	15	5
562	Glutathione reductase	GLR1	53	5
563	Protein TOS1	TOS1	48	4
564	Mitochondrial zinc maintenance protein 1, mitochondrial	MZM1	14	3
565	Transaldolase	TAL1	37	5
566	Maintenance of telomere capping protein 4	MTC4	79	6
567	Minichromosome maintenance protein 5	MCM5	86	5
568	Manganese-transporting ATPase 1	SPF1	135	5
569	Histone deacetylase RPD3	RPD3	49	8
570	Probable kynurenine--oxoglutarate transaminase BNA3	BNA3	50	7
571	Uncharacterized transcriptional regulatory protein YLR278C		151	7
572	Uncharacterized protein YBR225W		101	7
573	Regulator of the glycerol channel 1;Activator of SKN7 protein 10	RGC1;ASK10	120	4
574	Autophagy-related protein 20	ATG20	73	6
575	Putative aldehyde dehydrogenase-like protein YHR039C	MSC7	71	5
576	Uncharacterized protein YLR290C, mitochondrial	YLR290C	32	7
577	Putative DNA helicase INO80	INO80	171	8
578	Broad-range acid phosphatase DET1	DET1	39	5
579	Glutamate--tRNA ligase, mitochondrial	MSE1	62	8
580	Mitochondrial import inner membrane translocase subunit TIM23	TIM23	23	4
581	54S ribosomal protein MRP49, mitochondrial	MRP49	16	4
582	ATPase expression protein 2, mitochondrial	AEP2	68	10
583	Cytochrome B translational activator protein CBS2	CBS2	45	3
584	Heat shock protein SSA2	SSA2	69	4
585	Probable 2-?methylcitrate dehydratase	PDH1	58	5
586	Protein KES1	KES1	49	5
587	Acetyl-CoA carboxylase, mitochondrial;Biotin carboxylase	HFA1	259	9
588	Protein FMP52, mitochondrial	FMP52	25	5
589	Chorismate synthase	ARO2	41	5
590	54S ribosomal protein L27, mitochondrial	MRPL27	16	3
591	J-type co-chaperone JAC1, mitochondrial	JAC1	22	3
592	54S ribosomal protein L25, mitochondrial	MRPL25	19	5
593	Bromodomain-containing factor 1	BDF1	77	7
594	Serine/threonine protein kinase KIN1	KIN1	120	7
595	Protein EAP1	EAP1	70	5
596	ABC transporter ATP-binding protein ARB1	ARB1	68	8
597	Histidine--tRNA ligase, mitochondrial	HTS1	58	5
598	DNA repair and recombination protein RAD26	RAD26	125	7
599	Nitrogen permease regulator 3	NPR3	130	7
600	DNA replication licensing factor MCM4	MCM4	105	7
601	60S ribosomal protein L35-B;60S ribosomal protein L35-A	RPL35B;RPL35A	14	3
602	Chromatin structure-remodeling complex subunit RSC1	RSC1	107	4
603	Serine/threonine-protein kinase HAL5	HAL5	95	6
604	Nucleoporin NUP192	NUP192	192	7
605	Transcriptional adapter 2	ADA2	51	3
606	Epsin-5	ENT5	47	6
607	Protein translocation protein SEC63	SEC63	75	6
608	Mitochondrial transcription factor 1	MTF1	40	5
609	40S ribosomal protein S12	RPS12	15	5
610	37S ribosomal protein MRP17, mitochondrial	MRP17	15	3
611	ATP-dependent RNA helicase SUV3, mitochondrial	SUV3	84	4
612	Fumarate reductase 2	OSM1	55	5
613	BTB/POZ domain-containing protein YLR108C		56	7
614	Mitochondrial distribution and morphology protein 38	MDM38	65	5
615	Glutamine--fructose-6-phosphate aminotransferase [isomerizing]	GFA1	80	7
616	Dolichyl-phosphate-mannose--protein mannosyltransferase 1	PMT1	93	5
617	Bifunctional purine biosynthetic protein ADE5,7	ADE5,7	86	5
618	Mitochondrial chaperone BCS1	BCS1	51	3
619	Structural maintenance of chromosomes protein 3	SMC3	141	6
620	Stress response protein NST1	NST1	142	3
621	Uncharacterized ABC transporter ATP-binding protein YDR061W		61	6
622	Seventh homolog of septin 1	SHS1	63	5
623	Casein kinase I homolog 3	YCK3	60	5
624	Probable serine/threonine-protein kinase COQ8, mitochondrial	COQ8	57	6
625	Altered inheritance of mitochondria protein 46, mitochondrial	AIM46	34	6
626	Isoleucine--tRNA ligase, mitochondrial	ISM1	116	7
627	Coatomer subunit beta	SEC26	109	5
628	Nuclear migration protein NUM1	NUM1	313	5
629	Elongation factor 1-?gamma 2	TEF4	47	7
630	CCR4-NOT transcriptional complex subunit CAF120	CAF120	118	3
631	Mitochondrial outer membrane protein OM45	OM45	45	6
632	Protein phosphatase 2C homolog 7, mitochondrial	PTC7	38	5
633	UPF0674 endoplasmic reticulum membrane protein YNR021W		47	4
634	Glucose-signaling factor 2	GSF2	46	6
635	Sensitivity to high expression protein 10	SHE10	67	2
636	Protein SSP120	SSP120	27	5
637	High-affinity glucose transporter HXT2	HXT2	60	2
638	Glutamate--tRNA ligase, cytoplasmic	GUS1	81	6
639	rRNA 2-?O-methyltransferase fibrillarin	NOP1	34	3
640	DNA-directed RNA polymerase I subunit RPA135	RPA135	136	7
641	H/ACA ribonucleoprotein complex subunit 4	CBF5	55	5
642	Polyamine N-?acetyltransferase 1	PAA1	22	8
643	Mitochondrial inner membrane i-?AAA protease supercomplex subunit MGR1	MGR1	47	5
644	Dolichyl-diphosphooligosaccharide--protein glycosyltransferase subunit 1	OST1	54	3
645	60S ribosomal protein L1-B;60S ribosomal protein L1-A	RPL1B;RPL1A	24	3
646	GTP-binding protein YPT1	YPT1	23	3
647	Tricalbin-2	TCB2	133	7
648	37S ribosomal protein YMR-31, mitochondrial	YMR31	14	4
649	3-isopropylmalate dehydratase	LEU1	86	7
650	Negative regulator of sporulation PMD1	PMD1	195	6
651	Heat shock protein homolog SSE1	SSE1	77	8
652	Mitochondrial protein import protein ZIM17	ZIM17	20	4
653	1,3-beta-glucan synthase component FKS1	FKS1	215	6
654	DNA replication licensing factor MCM6	MCM6	113	6
655	Serine/threonine-protein kinase KIN3	KIN3	51	5
656	Mitochondrial clpX-like chaperone MCX1	MCX1	58	6
657	Tricalbin-1	TCB1	134	3
658	Transketolase 1	TKL1	74	7
659	T-complex protein 1?subunit epsilon	CCT5	62	4
660	Adenosylhomocysteinase	SAH1	49	4
661	DNA topoisomerase 1	TOP1	90	4
662	Adenosine kinase	ADO1	36	3
663	Mannose-1-phosphate guanyltransferase	PSA1	40	6
664	Reduced viability upon starvation protein 161	RVS161	30	3
665	Threonine--tRNA ligase, mitochondrial	MST1	54	5
666	Altered inheritance of mitochondria protein 24, mitochondrial	AIM24	44	7
667	Nucleoporin NUP157	NUP157	157	4
668	Cytochrome P450 61	ERG5	61	5
669	GTP-binding protein YPT52	YPT52	26	2
670	54S ribosomal protein L6, mitochondrial	MRPL6	24	4
671	NADP-specific glutamate dehydrogenase 1	GDH1	50	7
672	Actin-related protein 4	ARP4	55	4
673	Pentafunctional AROM polypeptide	ARO1	175	8
674	ATP synthase assembly factor FMC1, mitochondrial	FMC1	18	3
675	Phosphatidylinositol 4,5-bisphosphate-binding protein SLM1	SLM1	78	5
676	Replication factor C subunit 5	RFC5	40	4
677	Trans-acting factor D	RAF1	21	4
678	CCA tRNA nucleotidyltransferase, mitochondrial	CCA1	61	6
679	Tricarboxylate transport protein	CTP1	32	5
680	3-hydroxy-3-methylglutaryl-coenzyme A reductase 2	HMG2	116	4
681	10 heat shock protein, mitochondrial	HSP10	11	4
682	Cell wall protein YJL171C		43	4
683	E3 ubiquitin-protein ligase RSP5	RSP5	92	4
684	DNA-directed RNA polymerase II subunit RPB1	RPO21	192	6
685	ATPase synthesis protein 25, mitochondrial	ATP25	70	4
686	Dynamin-like GTPase MGM1, mitochondrial	MGM1	99	4
687	MEMO1 family protein YJR008W		38	8
688	54S ribosomal protein IMG2, mitochondrial	IMG2	16	3
689	Reduced viability upon starvation protein 167	RVS167	53	3
690	Protein SEY1	SEY1	89	4
691	1,3-beta-glucanosyltransferase GAS1	GAS1	60	3
692	ATPase expression protein 1, mitochondrial	AEP1	60	5
693	Protein TOM71	TOM71	72	8
694	54S ribosomal protein L36, mitochondrial	MRPL36	20	3
695	Uncharacterized membrane protein YGR149W		52	3
696	Mitochondrial escape protein 2	YME2	97	4
697	13 ribonucleoprotein-associated protein	SNU13	14	2
698	S-adenosylmethionine synthase 1;S-adenosylmethionine synthase 2	SAM1;SAM2	42	2
699	Arginine--tRNA ligase, mitochondrial	MSR1	74	7
700	Uncharacterized mitochondrial hydrolase FMP41	FMP41	29	3
701	Alanine--glyoxylate aminotransferase 1	AGX1	42	4
702	Endopolyphosphatase	PPN1	78	4
703	Protein PET54	PET54	35	3
704	Nuclear polyadenylated RNA-binding protein 4	HRP1	60	3
705	Thiosulfate sulfurtransferase RDL1, mitochondrial	RDL1	15	2
706	Mitochondrial import receptor subunit TOM20	TOM20	20	6
707	T-complex protein 1?subunit gamma	CCT3	59	4
708	Putative cysteine synthase	YGR012W	43	3
709	Alpha-mannosidase	AMS1	125	5
710	Magnesium-activated aldehyde dehydrogenase, cytosolic	ALD6	54	5
711	Transcription initiation factor TFIID subunit 5	TAF5	89	4
712	ADP-ribosylation factor GTPase-activating protein effector protein 1	AGE1	54	5
713	Intermediate cleaving peptidase 55	ICP55	58	4
714	Sphingoid long chain base kinase 5	LCB5	78	6
715	Oxysterol-binding protein homolog 6	OSH6	52	2
716	Mitochondrial inner membrane i-?AAA protease supercomplex subunit MGR3	MGR3	58	5
717	Cyclin-dependent kinase 1;Cyclin-dependent protein kinase PHO85	CDC28;PHO85	34	4
718	Chromo domain-containing protein 1	CHD1	168	4
719	NADPH dehydrogenase 2	OYE2	45	5
720	Partitioning protein REP2	REP2	33	3
721	Exoribonuclease II, mitochondrial	DSS1	111	5
722	DNA-directed RNA polymerases I and III subunit RPAC1	RPC40	38	3
723	Probable NADPH:adrenodoxin oxidoreductase, mitochondrial	ARH1	56	6
724	Tryptophan synthase	TRP5	77	5
725	Elongator complex protein 5	IKI1	35	1
726	Mitochondrial outer membrane protein IML2	IML2	83	5
727	DNA-binding protein RAP1	RAP1	92	3
728	Replication factor C subunit 3	RFC3	38	5
729	Pleiotropic ABC efflux transporter of multiple drugs	PDR5	170	4
730	Casein kinase II subunit alpha	CKA2	39	5
731	Replication factor C subunit 4	RFC4	36	4
732	54S ribosomal protein L50, mitochondrial	MRPL50	16	2
733	Nucleoporin NUP57	NUP57	57	3
734	DNA mismatch repair protein MSH6	MSH6	140	4
735	CAAX prenyl protease 1	STE24	52	3
736	Mitochondrial FAD-linked sulfhydryl oxidase ERV1	ERV1	22	2
737	DNA-directed RNA polymerase I subunit RPA49	RPA49	47	6
738	Nucleosome assembly protein	NAP1	48	4
739	Altered inheritance of mitochondria protein 23, mitochondrial	AIM23	41	3
740	SED5-binding protein 2	SFB2	99	5
741	Obg-like ATPase homolog	YLF2	46	3
742	DNA-directed RNA polymerase II subunit RPB2	RPB2	139	5
743	Sister chromatid cohesion protein 2	SCC2	171	4
744	Protein TAX4	TAX4	69	4
745	Ubiquitin-like protein MDY2	MDY2	24	3
746	Phosphatidylinositol transfer protein SFH5	SFH5	34	2
747	DNA replication licensing factor MCM7	MCM7	95	5
748	Putative 2-?hydroxyacyl-CoA lyase	YEL020C	61	4
749	Mitochondrial intermembrane space import and assembly protein 40	MIA40	45	2
750	Nitrogen permease reactivator protein	NPR1	86	2
751	Low-affinity glucose transporter HXT4;Probable glucose transporter HXT5	HXT4;HXT5	64	3
752	Glycerol 2-?dehydrogenase (NADP(+))	GCY1	35	2
753	DNA ligase 1	CDC9	82	3
754	Peroxiredoxin DOT5	DOT5	24	4
755	Putative mitochondrial translation system component PET127	PET127	93	4
756	Cytochrome b-?c1 complex subunit 7	QCR7	15	3
757	Transcriptional regulatory protein SIN3	SIN3	175	1
758	DBF2 kinase activator protein MOB1	MOB1	36	3
759	Frataxin homolog, mitochondrial;Frataxin homolog intermediate form	YFH1	19	3
760	RNA polymerase II transcriptional coactivator SUB1	SUB1	33	3
761	Cell division control protein 42	CDC42	21	4
762	Ribosome-recycling factor, mitochondrial	RRF1	26	3
763	Mannan polymerase complexes subunit MNN9	MNN9	46	4
764	Serine--tRNA ligase, mitochondrial	DIA4	50	4
765	GMP synthase [glutamine-hydrolyzing]	GUA1	58	3
766	Ergosterol biosynthetic protein 28	ERG28	17	3
767	Clathrin coat assembly protein AP180A	YAP1801	72	4
768	Alpha,alpha-trehalose-phosphate synthase [UDP-forming] 56 subunit	TPS1	56	5
769	NADP-dependent alcohol dehydrogenase 6	ADH6	40	3
770	ATP-dependent RNA helicase MRH4, mitochondrial	MRH4	63	3
771	DNA mismatch repair protein MSH1, mitochondrial	MSH1	109	3
772	FACT complex subunit SPT16	SPT16	119	4
773	Apoptosis-inducing factor 1	AIF1	41	4
774	Inositol phosphosphingolipids phospholipase C	ISC1	54	2
775	Probable mitochondrial transport protein FSF1	FSF1	35	4
776	Histone deacetylase HOS3	HOS3	79	3
777	Mitochondrial carrier protein RIM2	RIM2	42	2
778	Dolichyl-phosphate-mannose--protein mannosyltransferase 2	PMT2;PMT3	87	2
779	Translation initiation factor RLI1	RLI1	68	5
780	Protein LDB19	LDB19	90	2
781	Heterogeneous nuclear rnp K-?like protein 2	HEK2	42	3
782	GTP-binding protein YPT31/YPT8	YPT31	24	3
783	Inosine-5-monophosphate dehydrogenase 2 ;Putative inosine-5-monophosphate dehydrogenase 1	IMD2;IMD1	57	4
784	54S ribosomal protein L38, mitochondrial;54S ribosomal protein L34, mitochondrial	MRPL38	15	3
785	C-5 sterol desaturase	ERG3	43	5
786	Histone acetyltransferase GCN5	GCN5	51	3
787	Serine/threonine-protein kinase BUR1	SGV1	74	3
788	Eukaryotic translation initiation factor 2?subunit gamma	GCD11	58	3
789	Actin-related protein 3	ARP3	50	3
790	26S proteasome regulatory subunit RPN2	RPN2	104	3
791	Elongation factor 3B	HEF3	116	3
792	Protein NUD1	NUD1	94	3
793	SWI/SNF complex subunit SWI3	SWI3	93	3
794	Protein transport protein SEC1	SEC1	83	6
795	Protein disulfide-isomerase MPD1	MPD1	36	3
796	Signal recognition particle receptor subunit alpha homolog	SRP101	69	4
797	Suppressor of kinetochore protein 1	SKP1	22	3
798	Pre-mRNA-splicing factor ATP-dependent RNA helicase PRP43	PRP43	88	1
799	Protein MSP1	MSP1	40	3
800	Chitin biosynthesis protein CHS5	CHS5	74	4
801	Uncharacterized mitochondrial membrane protein FMP10	FMP10	28	3
802	Mitochondrial metalloendopeptidase OMA1	OMA1	39	2
803	Cytochrome c oxidase subunit 6, mitochondrial	COX6	17	2
804	Probable hydrolase NIT3	NIT3	33	2
805	Elongator complex protein 4	ELP4	51	2
806	Chromatin-remodeling complexes subunit NGG1	NGG1	79	3
807	PWWP domain-containing protein YLR455W	YLR455W	36	3
808	rRNA biogenesis protein RRP5	RRP5	193	5
809	Vacuolar transporter chaperone 2	VTC2	95	2
810	3-oxoacyl-[acyl-carrier-protein] synthase homolog	CEM1	48	3
811	Clathrin heavy chain	CHC1	187	4
812	Regulatory protein SIR3	SIR3	111	3
813	Protein SPT3	SPT3	39	3
814	Uncharacterized protein YOR385W	YOR385W	34	3
815	Guanine nucleotide-binding protein subunit beta 2	GPB2	99	3
816	Long chronological lifespan protein 2	LCL2	15	1
817	Transcription factor tau 131 subunit	TFC4	120	3
818	Mitochondrial nuclease	NUC1	37	4
819	Phosphatidylinositol transfer protein CSR1	CSR1	47	2
820	Cell cycle serine/threonine-protein kinase CDC5/MSD2	CDC5	81	2
821	FK506-binding protein 1	FPR1	12	3
822	Pumilio homology domain family member 4	PUF4	98	3
823	Actin-related protein 2	ARP2	44	3
824	Cytochrome c oxidase assembly protein COX15	COX15	55	3
825	Acyl-CoA desaturase 1	OLE1	58	2
826	SVP1-like protein 2	HSV2	51	4
827	Tubulin beta chain	TUB2	51	2
828	Amino-acid acetyltransferase, mitochondrial	ARG2	66	2
829	Nucleoporin NUP188	NUP188	189	1
830	Maintenance of telomere capping protein 5	MTC5	131	1
831	Protein JSN1	JSN1	120	2
832	Cysteine--tRNA ligase		88	4
833	Structural maintenance of chromosomes protein 1	SMC1	141	3
834	ISWI one complex protein 2	IOC2	93	2
835	Tryptophan--tRNA ligase, cytoplasmic	WRS1	49	2
836	Heat shock protein 104	HSP104	102	2
837	Ubiquitin carboxyl-terminal hydrolase RPN11	RPN11	34	3
838	tRNA modification GTPase MSS1, mitochondrial	MSS1	58	2
839	26S proteasome regulatory subunit RPN8	RPN8	38	2
840	Vacuolar protein sorting-associated protein 53	VPS53	95	2
841	5-formyltetrahydrofolate cyclo-ligase	FAU1	24	3
842	Cell division control protein 53	CDC53	94	5
843	Protein MET17;O-acetylhomoserine sulfhydrylase	MET17	49	2
844	DNA mismatch repair protein MSH2	MSH2	109	2
845	GTP-binding protein RHO3	RHO3	25	2
846	Peptide chain release factor 1, mitochondrial	MRF1	47	2
847	Putative transferase CAF17, mitochondrial	IBA57	57	2
848	Protein SOV1, mitochondrial	SOV1	105	2
849	Thioredoxin reductase 2, mitochondrial;Thioredoxin reductase 1	TRR2;TRR1	37	3
850	Diphosphoinositol polyphosphate phosphohydrolase DDP1	DDP1	22	3
851	Target of rapamycin complex 1?subunit KOG1	KOG1	178	3
852	ISWI one complex protein 3	IOC3	91	2
853	Mitochondrial GTPase 1	MTG1	42	2
854	Cystathionine beta-synthase	CYS4	56	1
855	Kynurenine 3-?monooxygenase	BNA4	52	3
856	Peroxisomal hydratase-dehydrogenase-epimerase	FOX2	99	3
857	Phosphomannomutase	SEC53	29	4
				


**#**	**Identified Proteins on Yfh1-His6 sample**	**Gene names**	**Molecular Weight**	**Total MS/MS Count**
1	Cysteine desulfurase, mitochondrial	NFS1	54	179
2	Alcohol dehydrogenase 3, mitochondrial	ADH3	40	147
3	Frataxin homolog, mitochondrial	FRDA	19	134
4	Iron sulfur cluster assembly protein 1, mitochondrial	ISU1	18	110
5	Glyceraldehyde-3-phosphate dehydrogenase 1	G3P1	36	100
6	V-type proton ATPase catalytic subunit A	VATA	119	96
7	Glyceraldehyde-3-phosphate dehydrogenase 3	G3P3	36	84
8	Casein kinase I homolog 2	KC12	62	76
9	Elongation factor 1-?alpha	EF1A	50	70
10	Peroxisomal acyl-coenzyme A thioester hydrolase 1	PTE1	40	61
11	Nicotinamidase	PNC1	25	61
12	Glyceraldehyde-3-phosphate dehydrogenase 2	G3P2	36	61
13	Repressible alkaline phosphatase	PPB	63	58
14	Myosin tail region-interacting protein MTI1	BBC1	128	55
15	Eukaryotic translation initiation factor 4E	IF4E	24	32
16	Nucleoporin POM152	PO152	152	31
17	Dihydrofolate synthetase	FOLD	48	30
18	Protein ISD11	ISD11	11	29
19	Putative glucokinase-2	EMI2	56	29
20	Uncharacterized WD repeat-containing protein YOL087C	YO087	125	24
21	Heat shock protein 60, mitochondrial	HSP60	61	24
22	Mitochondrial metal transporter 2	MMT2	52	20
23	Altered inheritance of mitochondria protein 32	AIM32	36	20
24	Acyl carrier protein, mitochondrial	ACPM	14	20
25	Cerevisin	PRTB	70	20
26	Iron sulfur cluster assembly protein 2, mitochondrial	ISU2	17	19
27	Putative transcription factor SEF1	SEF1	128	18
28	Protein ZPS1	ZPS1	28	18
29	mRNA-binding protein PUF3	PUF3	98	17
30	Alcohol dehydrogenase 1	ADH1	37	17
31	C-1-tetrahydrofolate synthase, mitochondrial	C1TM	106	17
32	Nicotinamide-nucleotide adenylyltransferase 1	NMA1	46	16
33	RNA-binding protein SRO9	SRO9	48	15
34	Factor arrest protein 8	FAR8	59	15
35	Threonine dehydratase, mitochondrial	THDH	64	15
36	Cap-associated protein CAF20	CAF20	18	14
37	Putative pterin-4-alpha-carbinolamine dehydratase	PHS	14	13
38	Uncharacterized protein YHR097C	YHP7	41	13
39	UTP--glucose-1-phosphate uridylyltransferase	UGPA1	56	13
40	Potassium-activated aldehyde dehydrogenase, mitochondrial	ALDH4	57	12
41	Cytochrome b2, mitochondrial	CYB2	66	12
42	Protein HBT1	HBT1	114	12
43	Serine hydroxymethyltransferase, cytosolic	GLYC	52	12
44	Carbon catabolite-derepressing protein kinase	SNF1	72	11
45	Nucleolar protein 3	NOP3	45	11
46	Intrastrand cross-link recognition protein	IXR1	68	10
47	(R,R)-butanediol dehydrogenase	BDH1	42	10
48	6,7-dimethyl-8-ribityllumazine synthase	RIB4	19	10
49	Serine/threonine-protein kinase HRK1	HRK1	86	9
50	Probable serine/threonine-protein kinase RTK1	RTK1	70	9
51	Ketol-acid reductoisomerase, mitochondrial	ILV5	44	8
52	Nicotinamide-nucleotide adenylyltransferase 2	NMA2	45	8
53	Heat shock protein SSC1, mitochondrial	HSP77	71	7
54	Protein OPY1	OPY1	38	7
55	Malate dehydrogenase, mitochondrial	MDHM	36	7
56	Acetyl-CoA hydrolase	ACH1	59	7
57	General negative regulator of transcription subunit 4	NOT4	65	7
58	ATP synthase subunit alpha, mitochondrial	ATPA	59	6
59	Citrate synthase, mitochondrial	CISY1	53	6
60	Uncharacterized protein YBL086C	YBI6	52	6
61	Acetylornithine aminotransferase, mitochondrial	ARGD	47	6
62	Serine/threonine-protein kinase YPK2/YKR2	YPK2	77	6
63	Succinate dehydrogenase [ubiquinone] flavoprotein subunit, mitochondrial	SDHA	70	6
64	Protein SOF1	DCA13	57	6
65	Protein PSP2	PSP2	66	6
66	Glutamate decarboxylase	DCE	66	6
67	Carboxypeptidase S	CBPS	65	6
68	Acetolactate synthase catalytic subunit, mitochondrial	ILVB	75	5
69	Serine hydroxymethyltransferase, mitochondrial	GLYM	54	5
70	Histone acetyltransferase ESA1	ESA1	53	5
71	Sorting nexin-3	SNX3	19	5
72	Protein SSD1	SSD1	140	5
73	Histone H2B.1	H2B1 (+1)	14	4
74	Elongation factor Tu, mitochondrial	EFTU	48	4
75	Pyruvate dehydrogenase E1 component subunit alpha, mitochondrial	ODPA	46	4
76	Histone H4	H4	11	4
77	Succinate dehydrogenase [ubiquinone] iron-sulfur subunit, mitochondrial	SDHB	30	4
78	Transcriptional activator/repressor MOT3	MOT3	54	4
79	Heat shock protein SSA1	HSP71	70	4
80	Uncharacterized protein YGR266W	YG5L	81	4
81	40S ribosomal protein S12	RS12	15	4
82	GTP cyclohydrolase 1	GCH1	28	4
83	3-oxoacyl-[acyl-carrier-protein] synthase homolog	CEM1	48	4
84	Nuclear migration protein NUM1	NUM1	313	4
85	Nuclear polyadenylated RNA-binding protein 4	HRP1	60	4
86	Altered inheritance of mitochondria protein 6	AIM6	44	4
87	Actin patches distal protein 1	APD1	36	4
88	54S ribosomal protein L17, mitochondrial	RM17	32	4
89	Serine/threonine-protein kinase CLA4	CLA4	94	4
90	Protein GTS1	GTS1	44	4
91	Pyruvate kinase 1	KPYK1	55	3
92	Cytochrome b-?c1 complex subunit 1, mitochondrial	QCR1	50	3
93	Mitochondrial metal transporter 1	MMT1	56	3
94	Enolase 2	ENO2	47	3
95	Histone H2A.1	H2A1 (+1)	14	3
96	Pyruvate dehydrogenase E1 component subunit beta, mitochondrial	ODPB	40	3
97	Uncharacterized protein JIP4	JIP4	99	3
98	Heat shock protein 42	HSP42	43	3
99	Ubiquitin carboxyl-terminal hydrolase 3	UBP3	102	3
100	Cytochrome c oxidase subunit 4, mitochondrial	COX4	17	3
101	Serine/threonine-protein kinase AKL1	AKL1	124	3
102	Zinc-regulated transporter 3	ZRT3	55	3
103	37S ribosomal protein S24, mitochondrial	RT24	37	3
104	Verprolin	VRP1	83	3
105	Serine/threonine protein kinase KIN1	KIN1	120	3
106	Low affinity vacuolar monovalent cation/H(+) antiporter	VNX1	103	3
107	Enhancer of polycomb-like protein 1	EPL1	97	3
108	40S ribosomal protein S3	RS3	27	3
109	Target of rapamycin complex subunit LST8	LST8	34	3
110	Probable ADP-ribose 1''-phosphate phosphatase YML087W	YMX7	32	3
111	Transcriptional activator/repressor GIS1	GIS1	99	3
112	ATP synthase subunit beta, mitochondrial	ATPB	55	2
113	Mitochondrial outer membrane protein porin 1	VDAC1	30	2
114	Fumarate hydratase, mitochondrial	FUMH	53	2
115	Cytochrome b-?c1 complex subunit 2, mitochondrial	QCR2	40	2
116	2-oxoglutarate dehydrogenase, mitochondrial	ODO1	114	2
117	GTPase-activating protein BEM2/IPL2	BEM2	245	2
118	Succinyl-CoA ligase [ADP-forming] subunit alpha, mitochondrial	SUCA	35	2
119	Protein MMF1, mitochondrial	MMF1	16	2
120	PAB1-binding protein 1	PBP1	79	2
121	Probable oxidoreductase AIM17	AIM17	53	2
122	2-isopropylmalate synthase	LEU1	?	2
123	40S ribosomal protein S14-A	RS14A (+1)	15	2
124	Homoaconitase, mitochondrial	LYS4	75	2
125	Cytochrome c1, heme protein, mitochondrial	CY1	34	2
126	Oxysterol-binding protein homolog 3	OSH3	114	2
127	Nitrogen permease regulator 3	NPR3	130	2
128	Oxysterol-binding protein homolog 2	OSH2	146	2
129	Sphingolipid long chain base-responsive protein PIL1	PIL1	38	2
130	Cobalt uptake protein COT1	COT1	48	2
131	Negative regulator of sporulation PMD1	PMD1	195	2
132	Putative 2-?hydroxyacid dehydrogenase YPL113C	YP113	45	2
133	UBP3-associated protein BRE5	BRE5	58	2
134	78 glucose-regulated protein homolog	GRP78	74	2
135	D-lactate dehydrogenase [cytochrome] 3	DLD3	55	2
136	Uncharacterized protein YBR225W	YB75	101	2
137	Clustered mitochondria protein 1	CLU	145	2
138	Alcohol dehydrogenase 4	ADH4	41	2
139	Biotin synthase, mitochondrial	BIOB	42	2
140	60S ribosomal protein L11-A	RL11A (+1)	20	2
141	Protein SCP160	SC160	135	2
142	40S ribosomal protein S28-A	RS28A (+1)	8	2
143	Chromatin modification-related protein YNG2	YNG2	32	2
144	Uncharacterized abhydrolase domain-containing protein YGR015C	YG19	38	2
145	Serine/threonine-protein kinase KIN2	KIN2	128	2
146	Protein WHI4	WHI4	71	2
147	Guanine nucleotide-binding protein subunit beta-like protein	GBLP	35	2
148	Protein LDB19	LDB19	90	2
149	40S ribosomal protein S8-A	RS8A	22	2
150	54S ribosomal protein L3, mitochondrial	RM03	44	2
151	Aconitate hydratase, mitochondrial	ACON	85	1
152	Phosphoglycerate kinase	PGK	45	1
153	Dihydroxy-acid dehydratase, mitochondrial	ILV3	63	1
154	Plasma membrane ATPase 1	PMA1	100	1
155	Ubiquitin-40S ribosomal protein S31	RS27A	17	1
156	ADP,ATP carrier protein 2	ADT2	34	1
157	Protein AST1	AST1	48	1
158	Aminomethyltransferase, mitochondrial	GCST	44	1
159	Acetolactate synthase small subunit, mitochondrial	ILV6	34	1
160	Enoyl-[acyl-carrier protein] reductase [NADPH, B-?specific], mitochondrial	ETR1	42	1
161	MAP-homologous protein 1	MHP1	155	1
162	Protein RMD9-like, mitochondrial	RMD9L	84	1
163	Suppressor protein MPT5	MPT5	95	1
164	10 heat shock protein, mitochondrial	CH10	11	1
165	Isocitrate dehydrogenase [NAD] subunit 2, mitochondrial	IDH2	40	1
166	Aldehyde dehydrogenase 5, mitochondrial	ALDH5	57	1
167	RHO1 GDP-GTP exchange protein 2	ROM2	153	1
168	Isocitrate dehydrogenase [NAD] subunit 1, mitochondrial	IDH1	39	1
169	Protein disulfide-isomerase	PDI	58	1
170	Mitochondrial peroxiredoxin PRX1	PRX1	29	1
171	Meiotic sister-chromatid recombination protein 3	MSC3	81	1
172	Homoisocitrate dehydrogenase, mitochondrial	LYS12	40	1
173	ATP synthase subunit 5, mitochondrial	ATPO	23	1
174	Epsin-5	ENT5	47	1
175	60S ribosomal protein L13-B	RL13B	23	1
176	Fructose-bisphosphate aldolase	ALF	40	1
177	NADH kinase POS5, mitochondrial	POS5	46	1
178	Flavohemoprotein	FHP	45	1
179	Branched-chain-amino-acid aminotransferase, mitochondrial	BCA1	44	1
180	Pyruvate decarboxylase isozyme 1	PDC1	61	1
181	ATP synthase subunit gamma, mitochondrial	ATPG	34	1
182	Cytochrome c iso-1	CYC1	12	1
183	tRNA N6-adenosine threonylcarbamoyltransferase, mitochondrial	QRI7	46	1
184	Hexokinase-1	HXKA	54	1
185	Phenylacrylic acid decarboxylase 1, mitochondrial	PAD1	27	1
186	ARS-binding factor 2, mitochondrial	ABF2	22	1
187	Single-stranded DNA-binding protein RIM1, mitochondrial	RIM1	15	1
188	Protein ZEO1	ZEO1	13	1
189	Mitochondrial protein import protein ZIM17	ZIM17	20	1
190	40S ribosomal protein S7-A	RS7A	22	1
191	Inosine-5'-monophosphate dehydrogenase 2	IMDH2	57	1
192	Protein SIS1	SIS1	38	1
193	Phosphoglycerate mutase 1	PMG1	28	1
194	Respiratory growth induced protein 2	RGI2	19	1
195	Methionyl-tRNA formyltransferase, mitochondrial	FMT	45	1
196	DnaJ homolog 1, mitochondrial	MDJ1	56	1
197	Regulatory protein MIG1	MIG1	56	1
198	DNA-binding protein RAP1	RAP1	92	1
199	Mitochondrial intermediate peptidase	PMIP	88	1
200	Nitrogen permease reactivator protein	NPR1	86	1
201	Amidophosphoribosyltransferase	PUR1	57	1

**Fig. 1:**
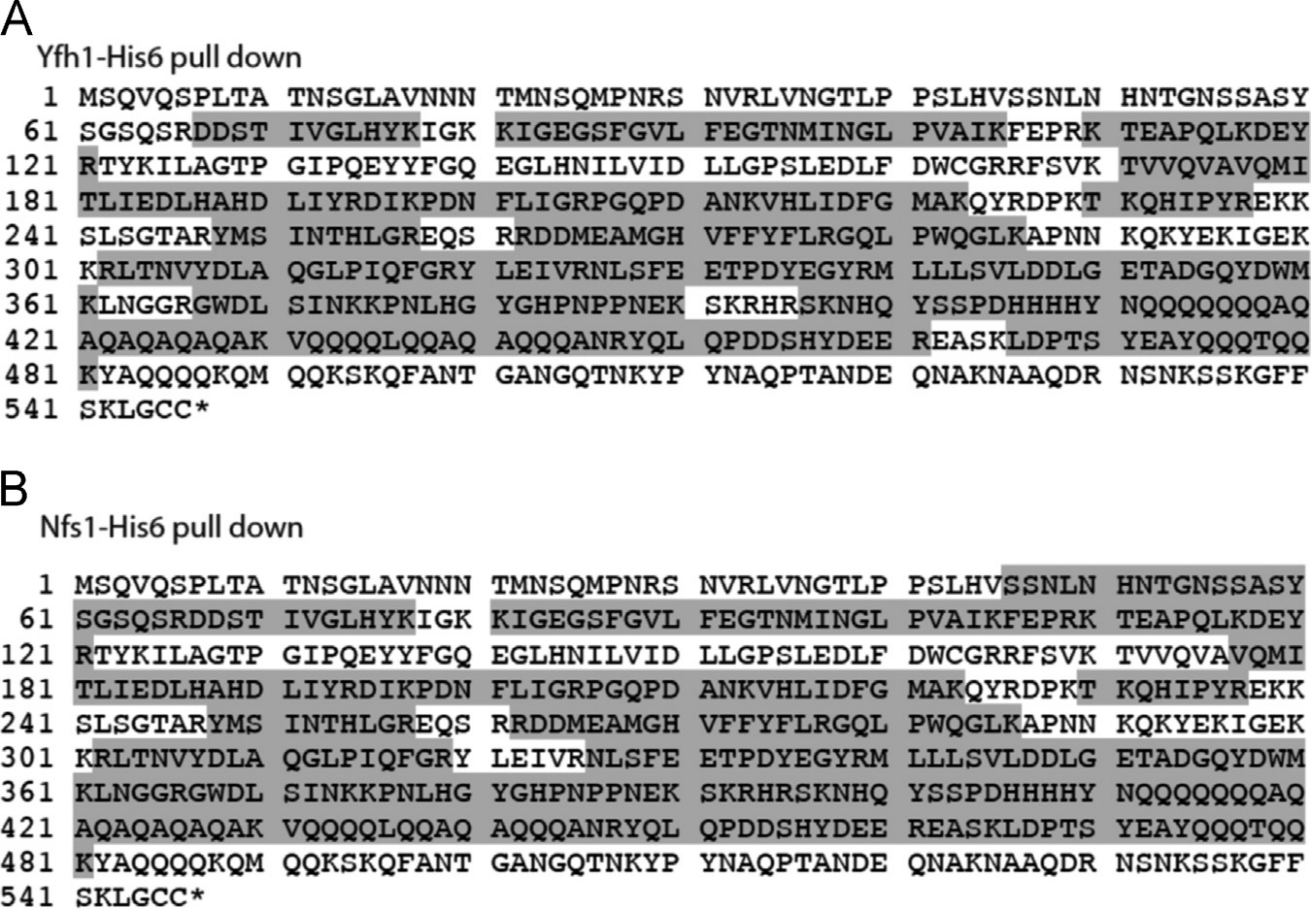
MS/MS peptide coverage of Yck2. Yck2 was detected by LC-MS/MS in pull-downs from mitochondria expressing Yfh1-His_6_ (A) or Nfs1-His_6_ (B). Gray boxes represent the identified peptides.

Yck2 was detected in pull-downs from mitochondria (Fig. 1).

**Fig. 2:**
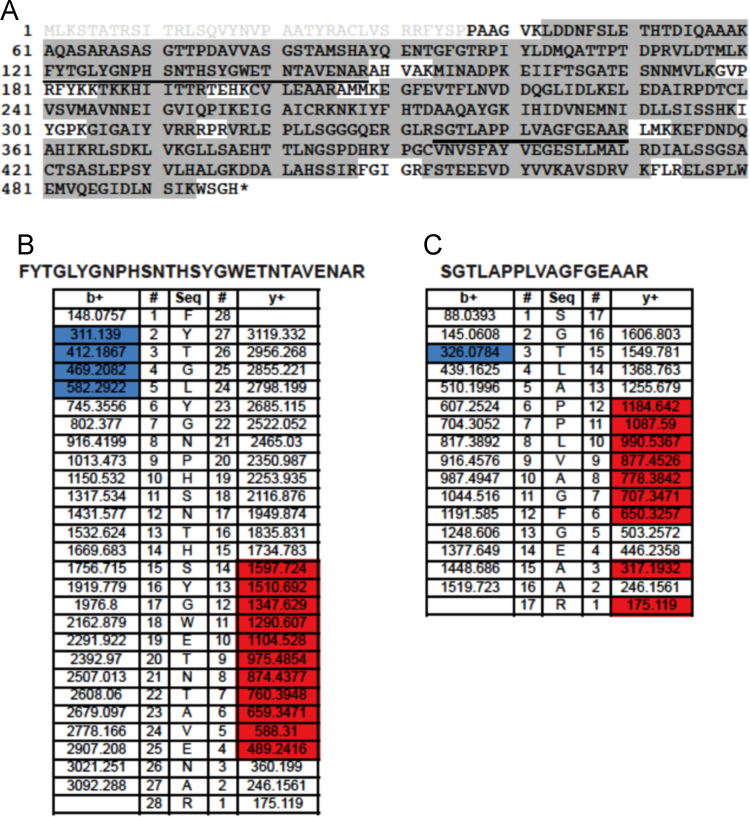
Nfs1 phosphopeptides identified by LC-MS/MS in experiment 1. The mature Nfs1-His_6_/Isd11 was recovered from *E. coli* co-expressing the two proteins. (A) Nfs1 protein coverage is showed by gray shading, and the predicted transit peptide is indicated by gray lettering. The phosphorylated peptides are underlined in the sequence. (B) Fragment ions from the peptide FYTGLYGNPHSNTHSYGWETNTAVENAR and (C) SGTLAPPLVAGFGEAAR are presented, where b ions are colored in blue and y ions in red.

**Fig. 3:**
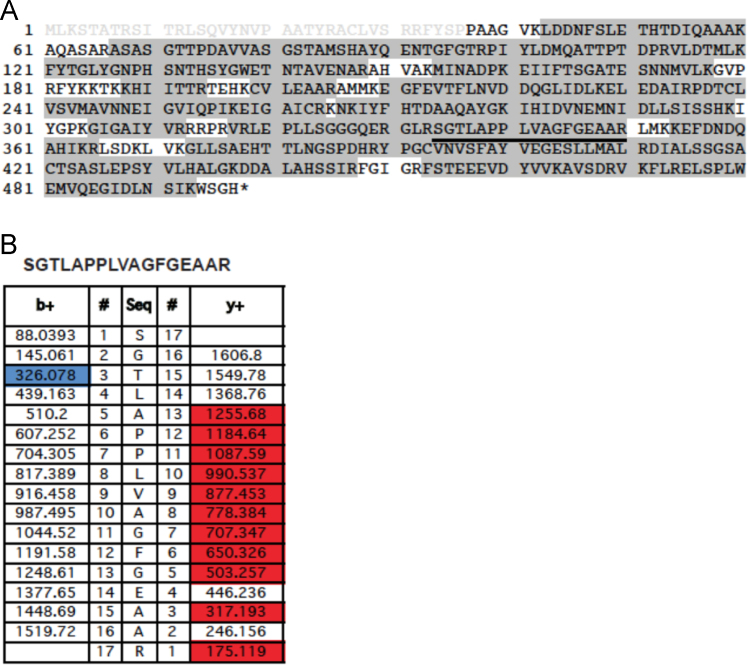
Nfs1 phosphopeptides identified by LC-MS/MS in experiment 2. Nfs1-His_6_ was recovered from isolated yeast mitochondria. (A) Nfs1 protein coverage is showed by gray shading, and the predicted transit peptide is indicated by gray lettering. The phosphorylated peptide is underlined in the sequence. (B) Fragment ions from the peptide SGTLAPPLVAGFGEAAR, where b ions are colored in blue and y ions in red.

**Fig. 4:**
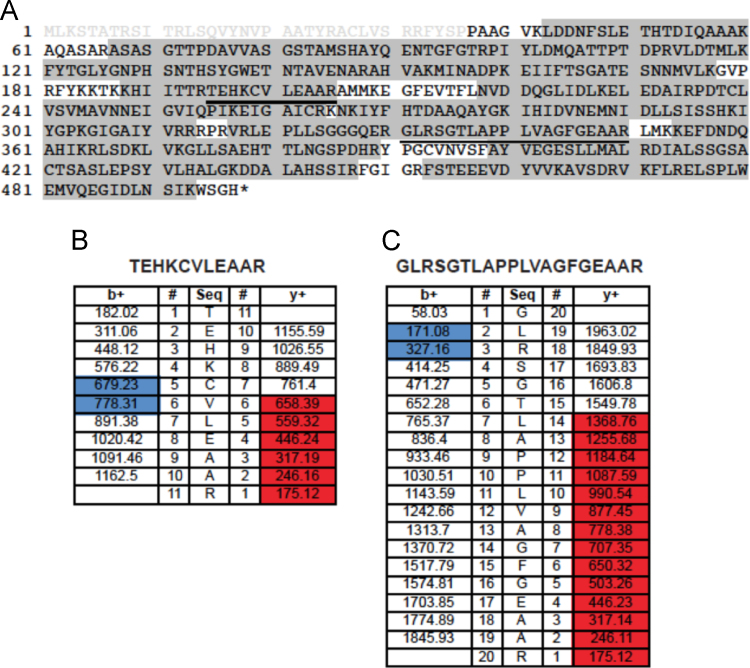
Nfs1 phosphopeptides identified by LC-MS/MS in experiment 3. Nfs1-His_6_/Isd11 was purified from *E. coli* and phosphorylated in the presence of the Yfh1_His6_ pull-down fraction from yeast mitochondria. (A) Nfs1 protein coverage is showed by gray shading, and the predicted transit peptide is indicated by gray lettering. The phosphorylated peptides are underlined in the sequence. Fragment ions from the peptides (B) TEHKCVLEAAR and (C) GLRSGTLAPPLVAGFGEAAR are shown, where b ions are colored in blue and y ions in red.

Nfs1 phosphopeptides identified by MS/MS in three independent experiments. Experiment 1. Nfs1_His6_/Isd11 purified in *E. coli* was purified (Fig. 2); Experiment 2. Nfs1_His6_ purified from yeast mitochondria (Fig. 3); Experiment 3. Nfs1_His6_/Isd11 purified from *E. coli*, exposed to ATP and a kinase active fraction from mitochondria (Fig. 4) [1].

## 2 Experimental design, materials and methods

### 2.1 Recombinant protein purification

The pST39-∆36Nfs1-His_6_-Isd11 plasmid, was transformed into BL21 (Codon^+^) RIL cells (Amp^R^; Chl^R^). Overexpression was induced at 30 °C in the presence of 0.5 mM IPTG for 3 h. Recombinant proteins were purified under native conditions by affinity chromatography using Ni-NTA superflow agarose (Qiagen). Proteins were eluted with 50 mM TrisCl pH 7.5, 0.15 M NaCl, 10% glycerol, and 400 mM imidazole. Protein concentrations were estimated by A_280_ and confirmed by SDS-PAGE staining using BSA standards as reference. All proteins were stored at −80 °C (Fig. 1, Fig. 2, Fig. 3, Fig. 4).

### 2.2 Pull-downs of mitochondrial proteins

Mitochondrial sub-fractions were enriched from the Yfh1-His_6_ strain using affinity chromatography. Briefly, 175 mg of isolated mitochondria were suspended in 20 ml of 50 mM TrisCl pH 7.5, 80 mM KCl, 20 mM imidazole, 1 mM 2-mercaptoethanol, 1 mM PMSF, 10% glycerol (Lysis buffer 1). Cells were passed five times through a French Press (~1500–2000 psi) followed by centrifugation at 108,000x*g* for 30 min using the TLA 100.3 rotor. The supernatant was incubated with 500 μl Ni-NTA superflow agarose (Qiagen) for 1 h in a rotating chromatography column and washed with 20 ml 50 mM TrisCl pH 7.5, 80 mM KCl, 40 mM imidazole, 1 mM 2-mercaptoethanol, 1 mM PMSF, 0.1% Triton X-100, 10% glycerol. Proteins were eluted with 3 ml of 50 mM TrisCl pH 7.5, 80 mM KCl, 400 mM imidazole, 1 mM 2-mercaptoethanol, 1 mM PMSF, 10% glycerol (Elution buffer 1).

The Gal-Nfs1-His_6_ strain was used to purify endogenous cysteine desulfurase from yeast mitochondria. Mitochondria were isolated, and 40 mg was suspended in 3.2 ml of Lysis buffer 1. Samples were sonicated with a probe (3 times, 20 sec each, duty cycle of 50%, with 1 sec gap) and further centrifuged at 20,000x*g* for 30 min. The pellet was suspended in the same volume of Lysis buffer 1, sonicated and centrifuged as previously. Both supernatants were combined in a chromatography column and incubated with 125 μl Ni-NTA superflow agarose (Qiagen) for 1 hour while rotating end over end. Beads were washed with 10 ml of 50 mM TrisCl pH 7.5, 80 mM KCl, 40 mM imidazole, 1 mM 2-mercaptoethanol, 1 mM PMSF, 10% glycerol. Proteins were eluted with 600 μl of Elution buffer 1. For experiments for detection of phosphosites, phosphatase inhibitors PhosSTOP (Roche) were included in all buffers, and experiments were carried out on ice or at 4˙C. Proteins were stored in aliquots at −80 °C.

### 2.3 LC-MS/MS analyses and data processing

Liquid chromatography-tandem mass spectrometry (LC-MS/MS) analysis was performed by the Proteomics and Metabolomics Facility at the Wistar Institute using a Q Exactive HF mass spectrometer (ThermoFisher Scientific) coupled with a Nano-ACQUITY UPLC system (Waters). Samples were digested in-gel with trypsin and injected onto a UPLC Symmetry trap column (180 μm i.d.×2 cm packed with 5 μm C18 resin; Waters). For phosphopeptide enrichment, samples were digested in-solution and subjected to Titansphere® TiO (GL Sciences) purification prior to LC-MS/MS analysis. Tryptic peptides were separated by RP-HPLC on a BEH C18 nanocapillary analytical column (75 μm i.d.×25 cm, 1.7 μm particle size; Waters) using a gradient formed by solvent A (0.1% formic acid in water) and solvent B (0.1% formic acid in acetonitrile). Peptides were eluted at 250 nL/min for 5–28% B over 120 min, 28–40% B over 5 min, 40–90% B over 5 min, constant 90% B for 10 min before returning to 5% B over 2 min. A 30-min blank gradient was run between sample injections to minimize carryover. Eluted peptides were analyzed by the mass spectrometer set to repetitively scan m/z from 300 to 2000 in positive ion mode. The full MS scan was collected at 60,000 resolution followed by data-dependent MS/MS scans at 15,000 resolution on the 20 most abundant ions exceeding a minimum threshold of 20,000. Peptide match was set as preferred, exclude isotopes option, and charge-state screening were enabled to reject singly and unassigned charged ions. MS data were analyzed with MaxQuant 1.5.2.8 [2]. MS/MS spectra were searched against the *S. cerevisiae* UniProt protein database (January 2016) using full tryptic specificity with up to two missed cleavages, static carboxamidomethylation of Cys, and variable oxidation of Met, protein N-terminal acetylation, and phosphorylation on Ser, Thr, and Tyr. Modified peptides were required to have a minimum score of 40. Consensus identification lists were generated with false discovery rates of 1% at protein, peptide and site levels.
